# Development and testing of the situational judgement test to measure safety performance of healthcare professionals: An explorative cross‐sectional study

**DOI:** 10.1002/nop2.1119

**Published:** 2021-11-01

**Authors:** Lina Heier, Nikoloz Gambashidze, Judith Hammerschmidt, Donia Riouchi, Franziska Geiser, Nicole Ernstmann

**Affiliations:** ^1^ Institute for Patient Safety University Hospital Bonn Bonn Germany; ^2^ Center for Health Communication and Health Services Research Department for Psychosomatic Medicine and Psychotherapy University Hospital Bonn Bonn Germany; ^3^ Department for Psychosomatic Medicine and Psychotherapy University Hospital Bonn Bonn Germany

**Keywords:** acute care, healthcare professionals, patient safety, safety performance, situational judgement test

## Abstract

**Aim:**

To measure safety performance, situational judgement test, which is a method composed of job‐related situations, can be used. This study aimed to develop and test its psychometric properties by measuring the safety performance of healthcare professionals in German hospitals.

**Design:**

An explorative cross‐sectional study.

**Methods:**

A team of researchers, nurses and physicians developed seven items, which focus on different safety areas. Descriptive statistics were calculated for each item. Cronbach's alpha was calculated as an indication of internal consistency. Spearman's correlation between the items was evaluated as analysis of construct validity. A cross‐sectional survey with healthcare professionals in three German hospitals was conducted to test the developed instrument.

**Results:**

A total of 168 healthcare professionals participated (response rate: 39.1%). 70.2% were women, and 38.7%, 33.9%, 15.5% and 11.3% were registered nurses, nurses in training, physicians and other healthcare professionals respectively. The situational judgement test demonstrated an acceptable psychometric performance.

## 1 INTRODUCTION

Adverse events, with an 8%–12% occurrence in all hospitalizations in European countries, have a huge impact on patient mortality and morbidity (Vries et al., 2008). In recent decades, especially since the publishing of the report “To err is human: building a safer health system” in 1998 (Kohn et al., 2000), the importance of safety skills, safety performance and safety culture have become clear and more evident (Brasaitė et al., 2016; Christian et al., 2009; Kiesewetter et al., 2018; Okuyama et al., 2014; Waterson et al., 2019). The World Health Organization (WHO) has recently published a global action plan, which provides a direction for concrete actions to be taken by healthcare facilities, countries and WHO itself to implement World Health Assembly resolution WHA72.6 (World Health Organization, 2021). This resolution gives priority to patient safety as an important step in designing, operating and evaluating the performance of all healthcare systems (World Health Organization, 2021). Several strategies provide detailed steps, addressing safety culture and patient safety strategies in all clinical programmes (e.g., infection prevention, medication safety, safety and medical devices among other safety topics; World Health Organization, 2021). A number of survey instruments have been developed and used to measure safety culture, and a link between safety culture and safety performance in health care is emerging (Okuyama et al., 2018; Pronovost et al., 2003; Scott et al., 2003; Waterson et al., 2019). Hospitals, along other health institutions, are implementing measures of patient safety and improving strategies for patient safety culture, which reflects the individual and group values, attitudes, behaviour patterns, competencies and perceptions (Brier et al., 2015; Granel et al., 2020; Okuyama et al., 2018).

## 2 BACKGROUND

Registered nurses, nursing students and physicians are actively involved in improving this multidisciplinary and multi‐professional approach of safety performance. Along other skills and competencies, they need safety skills to recognize patient safety incidents (e.g. syringe labelling), work in a team, learn from errors and use problem‐solving techniques and practice development skills (Brasaitė et al., 2016; Kwiecień‐Jaguś et al., 2018; Lavoie et al., 2020; Tower et al., 2019; Willman et al., 2020). Nurses, as the largest healthcare professional group, have an extraordinary impact on patient safety and their safety performance influences quality of care, well‐being and health outcomes of their patients. They are a constant presence for the patient, interact with other HCP on a regular basis and are responsible for monitoring patients’ condition, understanding and communicating care processes and changes in patient condition (Peck Malliaris et al., 2021). Furthermore, healthcare professionals (HCPs) can become causes of near misses, adverse events and errors (Alsharari et al., 2021; Kiesewetter et al., 2018; Veloski et al., 2005). Research with focus on individual level and its impact on patient safety is rare, although these gaps in knowledge are currently being addressed by using alternative research designs; therefore, an increasing number of qualitative studies are being published (Granel et al., 2020; Manapragada et al., 2019; McNab et al., 2016). Another option to measure the safety performance on the individual level and its impact on patient safety is a situational judgement test (SJT). It is a method composed of challenging work‐related situations and different courses of action (Lievens & Motowidlo, 2016; Muck, 2013; Oostrom et al., 2015; Patterson et al., 2016b). Situations may be presented in verbal, video‐based or written formats and contain different options (answer possibilities) from which the study participant chooses the most appropriate response (Christian et al., 2010). They have a long history of use for employee or student selection, and scenarios, which typically describe a dilemma or problem requiring knowledge, skills and abilities, are being used (Christian et al., 2010). SJT provides a reliable and cost‐effective method for measuring non‐academic attributes that are significant for clinicians and other HCPs (Cousans et al., 2017; Patterson, Knight, et al., 2016; Patterson, Zibarras, et al., 2016). Validated and reliable SJTs are available for nursing and medical school assessment, recruitment and hiring and for job performance evaluation in general practice (Bledow & Frese, 2009; Cousans et al., 2017; Crook et al., 2011; McDaniel et al., 2001; Neal et al., 2018; Patterson et al., 2017; Patterson, Zibarras, et al., 2016). However, to the best of our knowledge, no validated SJT measuring the safety performance of HCPs exists.

### 2.1 Research question

The first objective of this study was to develop items describing safety‐relevant situations in routine health care and corresponding answer categories with possible courses of action. The second objective was to test the set of items in a sample of HCP to evaluate its validity and reliability.

## 3 THE STUDY

### 3.1 Design

An exploratory cross‐sectional study of HCPs working in German hospitals between July 2019 and March 2020 (Safety Performance of HCP project) was conducted, to pilot the newly developed SJT. The Safety Performance of HCP project is built upon the integrative model of workplace safety and focuses on safety performance as a construct of safety compliance and safety participation (Christian et al., 2009; Neal & Griffin, 2002). The study population consists of registered nurses, nursing students (last year of training) and physicians from three acute hospitals and two nursing schools in Germany. Risk managers, medical directors and nursing managers and headmasters of nursing schools were informed about the study via email and/or personal contact at the ward. Each participant received a questionnaire using an online survey system or a paper–pencil format. Data collection in each organization lasted for approximately 6 weeks, and participants were reminded every other week.

### 3.2 Methods

#### 3.2.1 SJT development

To ensure content validity and internal consistency, the development of SJT items in this study followed the recommendation for SJT development in the medical training of Patterson and Zibarras, et al., 2016). Item development follows a process consisting of six sequential steps (Patterson, Zibarras, et al., 2016). In the first step of SJT development in the present study, a team of researchers, academic nurses (registered nurses who are working in academics) and physicians, all working in the field of patient safety, started with a safety performance role analysis of physicians, registered nurses and nursing students in acute medical care. Key attributes and competencies of different healthcare professions regarding safety compliance, safety participation and safety knowledge were gathered and analysed (Patterson, Zibarras, et al., 2016). This ensures that the content and situations of the items reflect everyday working scenarios (Patterson, Zibarras, et al., 2016). The results were seven different safety situations, which reflect everyday working areas (hygiene, workplace safety, patient identification, patient involvement, prophylaxis, infection prevention and communication).

During the second step, the test construction was specified: all SJT items were knowledge based, with a multiple‐choice answer possibility (three answers per item), provided in a pencil–paper format and an online survey system. The SJT items were introduced with a brief, two‐to‐three‐sentence situation description, followed by an instruction to choose the three out of 10 actions that best reflect the participant's behaviour in real life.

Step 3 is the actual item development and first reviews, to make sure the scenarios and responses are realistic, appropriate and plausible (Patterson, Zibarras, et al., 2016). On the basis of the seven safety situations, which were gathered in step 1, seven items with 10 response options each were developed, representing different safety topics (hygiene, workplace safety, patient identification, patient involvement, prophylaxis, infection prevention and communication). The seven different situations and answer options depict daily working situation in acute care and should be equally relevant for all HCPs. An example item is presented in Table 1.

**TABLE 1:** Example of a SJT item: patient identification

Situation	A patient (65 years old, open fracture after a bicycle fall) comes to the emergency centre and receives acute medical care. When transferring to the radiology, it is noticeable that the patient chart has a different name than the patient
Filling instructions	What corresponds most closely to your reaction? Please bear in mind how you would really react in your daily work. It is not a question of knowledge; it is an assessment of your actual behaviour Choose three most appropriate actions you would take in this situation
Answer options	Actively ask the patient for his full name and date of birth Search the emergency centre for the right patient chart Inform colleagues in radiology about the lack of patient identification Ask the patient about his previous treatment Explain the situation in the team and address the relevance of patient identification Make sure patient is wearing patient bracelet and this is the right one Contact the responsible physician to see if he has performed a patient identification Write a CIRS message Inform and calm the patient Don't tell the patient so he won't be worried

Note

CIRS – Critical Incidence Reporting System, a reporting system to systematically collect the hospital‐wide information about patient safety relevant incidents for organizational learning and continuous improvement.

To develop the scoring system (the fourth step in the SJT development), an expert group of HCPs was asked to choose the three most appropriate actions in terms of safety performance for each of the situations (Bergmann et al., 2006). The expert group consisted of physicians (*n* = 4), nurses (*n* = 8), nursing students (*n* = 10) and researchers (with a background in patient safety and health services research; *n* = 6). The answers provided by the expert group were analysed, and a safety performance score (SPS) was developed. The answer options, which were chosen by >40% of the experts, were assigned 2‐point, followed by 1‐point (15%–40%) and 0‐point (<15%) answers. On the basis of the instruction to choose three options, it is possible to achieve a score between 0 and 6 points. SPS was calculated as the average of available seven items, also ranging between 0 and 6 points. SPS scores ≤2.5, between 2.5–4.5 and between 4.5–6.0 were considered basic, advanced and expert safety performance respectively.

The SJT was piloted with a survey among HCPs in step 5 (please see section study design and setting).

#### 3.2.2 Analysis

In the sixth and last step, the development of an SJT to measure the safety performance of HCP was finalized with a psychometric analysis (Patterson, Zibarras, et al., 2016). Descriptive statistics were calculated for each SJT item (frequencies, means, standard deviations and minimum and maximum scores). Cronbach's alpha, as an indication of internal consistency of the instrument, was calculated (Field, 2018; Hair, 2010). Spearman's correlation between the SJT items was evaluated as an analysis of construct validity. Low‐to‐moderate positive correlations were expected because all items were considered to be measuring constructs related to safety performance.

### 3.3 Ethics

The study followed the ethical principals in accordance with the Declaration of Helsinki. The participants received written information about the study and an informed consent form together with privacy policy documents were attached to the questionnaire. The study was given ethical approval by a local ethical review board (number: 075/19).

Due to the sensitive topic of measuring safety performance, all professions were precisely informed about the protection of their person and data as well as the publication of the results. It was ensured that participation is completely anonymous and that no conclusions can be drawn about individuals or teams. Nurses were informed about the study in team meetings, physicians with an information letter and students with an introductory session on patient safety. In this way, all uncertainties and questions could be asked and clarified promptly. In addition, members of the project team visited the clinics every 14 days to answer any questions or concerns about patient safety or safety performance.

## 4 RESULTS

### 4.1 Study sample

Thirteen departments from three hospitals and two nursing schools were included in the study. A total of 430 HCPs were invited to participate. The response rate was 39.1% (*N* = 168). Of the participants, 70.2% were women, and 53.0% were <31 years old. In addition, 38.7%, 33.9%, 15.5% and 11.3% were registered nurses, nurses in training, physicians and other HCPs respectively. Furthermore, of the participants, 14.9% reported of having leadership roles. Details of the study sample are presented in Table 2.

**TABLE 2:** Sociodemographic data of the sample (*N* = 168)

	*n*	%
Gender		
Female	118	70.2
Male	50	29.8
Age (year)		
<30	89	53.0
31–40	31	18.5
41–50	24	14.3
>50	23	13.7
Profession		
Physician	26	15.5
Nurse	65	38.7
Nursing student	57	33.9
Others	19	11.3
Leadership role		
Yes	25	14.9
No	139	82.7
Work experience		
<3 months	2	1.2
>3 months <1 year	1	0.6
1 to 5 years	81	48.2
>5 years	82	48.8
Period of employment		
<3 months	4	2.4
>3 months <1 year	6	3.6
1 to 5 years	92	54.8
>5 years	65	38.7

### 4.2 Data processing

The study participants have not frequently chosen exactly three answers; in individual cases, up to seven answers were selected. To maintain consistency of scoring, the cases with >3 selected answers were considered invalid and were treated as missing in the analysis. Detailed numbers of missing and invalid cases are presented in Table 3.

**TABLE 3:** Descriptive statistics of seven test items and of overall Safety Performance Score (SPS)

	Missing cases	Invalid cases (>3 answers)	Used cases	Mean score	Standard deviation	Min	Max
SPS			148	4.43	0.72	1.86	5.57
Item 01 (Infection Prevention)	10	15	143	5.10	1.19	1.00	6.00
Item 02 (Communication)	10	2	156	4.37	1.33	1.00	6.00
Item 03 (Patient Identification)	14	21	133	4.39	1.06	0.00	6.00
Item 04 (Patient Involvement)	20	8	140	4.01	1.38	0.00	6.00
Item 05 (Prophylaxis)	19	7	142	4.47	1.27	1.00	6.00
Item 06 (Workplace Safety)	20	4	144	4.12	1.45	0.00	6.00
Item 07 (Hygiene)	20	5	143	4.73	1.27	0.00	6.00

### 4.3 SJT

The overall mean of all items was 4.38 (standard deviation, 0.75; range, 1.86–5.57). Item 03 (Patient Identification) and item 01 (Infection Prevention) had the highest numbers of invalid cases (21 and 17 cases respectively), which means that 21 and 17 participants selected four or more answer possibilities, instead of three. Moreover, item 01 (Infection Prevention) had the highest mean score of 5.09. Item 04 (Patient Involvement) had the lowest mean score of 3.93. All other items resulted in mean scores >4.0 (Expert Safety Performance). The results of the descriptive analysis are detailed in Table 3.

### 4.4 Internal consistency and construct validity

We included complete cases for analyses of internal consistency and construct validity (*N* = 111). The items of the SJT demonstrated Cronbach's alpha of 0.57. Moderate positive correlations were found among the seven variables in the study. Item 01 (Infection Prevention) showed significant correlation with item 02 (Communication) and item 06 (Workplace Safety). Item 02 (Communication) correlated significantly with item 06 (Workplace Safety) as well as item 07 (Hygiene) and item 03 (Patient Identification). Item 04 (Patient Involvement) correlated with item 06 (Workplace Safety). Item 05 (Prophylaxis) also correlated with item 06 (Workplace Safety). The least correlated items were item 04 (Patient Identification) and item 05 (Prophylaxis). No items were redundant, and there was no excessive correlation between the items (Spearman's rho > 0.85). All correlations are presented in Table 4.

**TABLE 4:** Spearman's correlations between items

	SPS	01	02	03	04	05	06	07
Item 01 (Infection Prevention)	0.49**	1.00	0.23*	0.18^+^	0.13	0.08	0.28*	0.07
Item 02 (Communication)	0.56**		1.00	0.20*	−0.04	0.10	0.28*	0.19*
Item 03 (Patient Identification)	0.32**			1.00	0.00	−0.07	0.09	−0.01
Item 04 (Patient Involvement)	0.40**				1.00	0.08	0.20*	−0.03
Item 05 (Prophylaxis)	0.38**					1.00	0.19*	0.14
Item 06 (Workplace Safety)	0.63**						1.00	0.04
Item 07 (Hygiene)	0.42*							1.00

Note

SPS: Safety Performance Score; Analysis with complete cases only (*N* = 111); Cronbach's alpha = 0.57.

*p <.05; **p <.001; ^+^ *p* =.06.

### 4.5 SPS

On the overall SPS, 56.8% of study participants reached scores ≥4.5, indicating an expert safety performance. 40.5% got an advanced SPS (2.5–4.5) and 2.7% a basic SPS (<2.5). On the single item level, 81.1% got an expert SPS on item 01 (Infection Prevention). More than half of participants got an expert SPS on item 02 (Communication) with 58.3%, item 05 (Prophylaxis) with 56.3% and item 07 (Hygiene) with 51.7%. Less than half of participants got expert SPS on item 03 (Patient Identification), item 04 (Patient Involvement) and item 06 (Workplace Safety).

**FIGURE 1:**
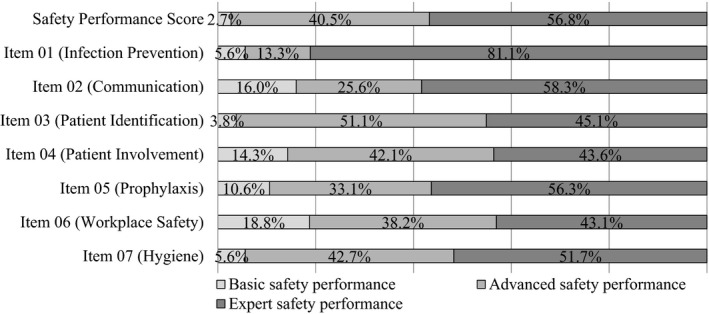
Basic, advanced and expert safety performance score on single item level as well as an overall safety performance score

Four items got a basic SPS ≥ 10%: item 06 (Workplace Safety) got the highest percentage of a basic SPS with 18.8%, followed by item 02 (Communication) with 16.0%, item 04 (Patient Involvement) with 14.3% and item 05 (Prophylaxis) with 10.6%. We report the distribution of the basic, advanced and expert SPS detailed in Figure 1.

## 5 DISCUSSION

This study demonstrated the development and testing of SJT for measuring the safety performance of HCPs working in acute care in Germany. To improve transparency, content validity and reliability in item development, the development of SJT items followed the recommendation of Patterson and colleagues (2016).

In this study, the newly developed instrument demonstrated an acceptable psychometric performance. The items included in the SJT were developed to cover a wide range of situations focusing on safety performance and relevant for most HCPs working in clinical settings. Majority of the participants were able to provide valid answers to all items. The situations were designed not to repeat, although designed to complement, each other and build a more comprehensive picture of safety performance at the frontline. This was reflected in positive but low correlations, with no significant negative correlations between items. In a meta‐analysis, the internal consistency coefficient of SJT items ranged from 0.43 to 0.94 (McDaniel et al., 2007). On the basis of this range, our instrument had acceptable internal consistency measured using Cronbach's alpha (0.57) for newly developed SJT with a diverse set of items (Catano et al., 2012). To further evaluate the reliability of the instrument, future studies should seek to establish test–retest reliability (Catano et al., 2012; Lievens et al., 2008; McDaniel et al., 2007).

Assessments of SPSs in different domains and subgroups have to be subjected to future studies. However, the findings of this study suggest an expert and advanced safety performance among frontline HCPs. Expert safety performance was pronounced for infection prevention, communication and prophylaxis. Basic safety performance was found for workplace safety and communication. Whether HCPs do not safely perform in certain areas (e.g. communication) or whether the items do not capture safety performance well will have to be clarified in future validation studies.

In a previous research, SJTs were used to measure several performance outcomes, for example, job performance (Chan & Schmitt, 2002; Lievens et al., 2008; McDaniel et al., 2001), personal initiative (Bledow & Frese, 2009) or job knowledge (Crook et al., 2011). With respect to job performance, research shows that SJTs seem to be a good predictor and should be as valid as those frequently used interviews and biographical measures (Chan & Schmitt, 2002; McDaniel et al., 2001). In this study, the newly developed SJT demonstrated an acceptable psychometric performance. However, whether the SJT developed and tested in this study is also a good predictor for safety performance remains an outstanding question and should be considered in future research.

### 5.1 Limitations

There are several methodological limitations to our research that should be considered when interpreting the results. Because of the exploratory character of this study, the results should be considered indicatory.

With regard to the development of the items, it should be noted that the development workshop was largely conducted by professionals with a nursing background, which may influence the item content and specific situations. For the expert scoring, not only experts in patient safety were selected but also other HCPs, who have no additional training in safety performance. If more experts had been involved in the scoring, the scoring system might have been chosen differently and consequently in different outcomes. Summarized, we cannot exclude possible limitations in the methodology, such as biases in rating and variations in how the study population understood the situations. The items with a high percentage of missing or excluded cases may indicate a higher item difficulty. Furthermore, order effects of SJT items are well known and should be considered while interpreting the results (Marentette et al., 2012).

It must also be noted that the topic of safety performance in acute care can be strongly influenced by social desirability and framing effects. Moreover, 14.9% of participants hold a leadership position that may influence SJT results. Supervisors can have a strong influence on patient safety as well as the safety performance of employees, what needs to be addressed when interpreting the results (Cavazotte et al., 2013; Ring & Fairchild, 2013). Future studies should take a closer look at the effect and differences of leadership positions and HCP as well as other hierarchical levels on the topic of safety performance and patient safety. In addition, responses may not reflect impacts of stress in patient safety‐related scenarios, which HCPs may experience at the frontline and influence their performance.

Furthermore, we acknowledge that our sample size was limited and that its composition can limit the external validity of our results. A modest response rate of 39.01% and 168 HCP in our study is a result of the convenience of our sampling approach and the proportions of the surveyed professions, which could cause selection bias. Similar study population sizes have been reported for SJT to measure hygiene competencies of HCP, among others (Heininger et al., 2021). In patient safety research, response rates of HCP under 50% are not uncommon (Robertson et al., 2015). No information was available for non‐respondents, which is a further limitation and should be taken into account while interpreting the trustworthiness of our study.

## 6 CONCLUSION

The explorative study presents the development and testing of SJT to measure the safety performance of HCPs working in acute care. The SJT demonstrated an acceptable psychometric performance and can be used to measure safety performance of HCPs in certain areas, such as hygiene, patient identification and infection prevention. Having only detailed knowledge is insufficient in order to work safe, the knowledge needs to be applied correctly across a multitude of situations (Heininger et al., 2021). Therefore, the SJT helps to identify specific safety gaps at the individual level of nurses, nurses in training and physicians, which thereby can be addressed for further interventions to improve patient safety. Further research is needed to answer questions about time effects in longitudinal research studies, construct validity, in particular, in comparison with other measurements, such as non‐participating observations.

## CONFLICT OF INTEREST

The authors have no conflict of interest to declare.

## AUTHOR CONTRIBUTIONS

All authors conceived and designed the study. Recruitment, data collection and data management with the assistance of DR, JH, FG and NE; data analysis with the assistance of NG and NE and manuscript draft, including tables and figures: LH. All authors reviewed the manuscript, provided comments and approved the final version.

## ETHICAL APPROVAL

The study was conducted according to the guidelines of the Declaration of Helsinki, and approved by the Ethics Committee of the Medical Faculty of the University of Bonn, Germany (Number: 075/19, 30 April 2019).

## Data Availability

The data that supports the findings of this study are available from the corresponding author upon reasonable request.
